# Medication Required: P*RN – Promoting Healthy Attitudes and Improved Access to Pornography in Wathwood Hospital

**DOI:** 10.1192/bjo.2024.410

**Published:** 2024-08-01

**Authors:** Rianne Parmar, Joanne Parry, Thomas Nicoll, James Dugan

**Affiliations:** 1 Sheffield Health and Social Care, Sheffield, United Kingdom; 2 Nottinghamshire Healthcare NHS Foundation Trust, Nottingham, United Kingdom

## Abstract

**Aims:**

The key aim of this project was to update and modernise the hospital procedure on how patients access pornographic material whilst detained at Wathwood Hospital. Within the update, we aimed to promote inclusivity and acceptance of all patients’ sexual identities as well as utilising the opportunity to emphasise healthy consenting sexual relationships.

Patients in Forensic Mental Health settings are often inpatients for a significant amount of time, with multiple restrictions imposed on their private and family life. Current policy allows patients to purchase pornography for private use in their own room. It must be material of the same nature available in a main street outlet. In practice, material is usually purchased in DVD format from the Amazon website and subsequently screened for suitability by Security staff and finally approval by the Responsible Clinician. There have been numerous incident reports involving the trading of pornographic material.

**Methods:**

Qualitative semi-structured group interviews (up to 5 people at a time) were conducted with patients in the medium-secure forensic services of Wathwood Hospital. They were recruited from the fortnightly Patient Forum. Anonymised questionnaires involving Likert scales and free text response spaces were also distributed at the Patient Forum. Data gathered investigated the percentage of patients who were aware of the current procedure, if they felt it worked well and what they thought the impact of accessing pornographic material might be. Staff were invited to complete a similar anonymised questionnaire, again considering their opinions on the positive or negative impacts of pornography for patients. In addition, we gathered data on whether there was a difference on the degree of comfort/discomfort about pornography, depending on whether the material involved opposite sex or same sex couples. In total, there were 40 survey participants.

**Results:**

Some key areas for concern were found, for example, only 17% of staff and 16% of patients thought the current policy works well despite 69% of staff and 84% of patients feeling it is a patient's right to access pornography. Free text and focus group feedback established many benefits to it. It was clear that there were some areas of difficulty in the hospital policy, which would benefit from being refreshed.

**Conclusion:**

Staff and patients overall feel that access to pornography is important for many of the patients. We identified areas for improvement in how this is accessed and a need to continually be considering the need to consider meeting the holistic needs for the patients.

